# Processing–Structure–Property Relationships in Polymer/Boron Nitride Nanotube Composite Fibers for Electronic Packaging Applications

**DOI:** 10.1021/acsanm.5c02920

**Published:** 2025-09-18

**Authors:** Casey L. Smith, Keenan J. Mintz, Kishor Gupta, Anita Garg, Laura Wilson, Satish Kumar

**Affiliations:** † 1372 Georgia Institute of Technology, School of Materials Science and Engineering, Atlanta, Georgia 30332, United States; ‡ University of Toledo, Mechanical Industrial and Manufacturing Engineering, Toledo, Ohio 43606, United States; § 53522 NASA Glenn Research Center, Cleveland, Ohio 44135, United States

**Keywords:** boron nitride nanotubes, polymer composite fibers, nanotube orientation, nanotube purification, dispersion methods, thermally conductive materials

## Abstract

Boron nitride nanotubes are promising materials for polymeric composites due to their electrical insulation and thermal conductivity properties. In this work, boron nitride nanotubes (BNNTs) are dispersed in a polymer solution that is then spun and drawn to make polymeric fibers with aligned bundles of long BNNTs. These polymer/BNNT fibers are then studied to determine the relationship among processing, structure, and properties. A sonication-centrifuge procedure was conducted to preserve longer BNNTs within the polymer structure and improve the alignment of BNNTs within the fiber. Herein, changes in the internal structure of PAN/BNNT fiber are mapped, highlighting the importance of the dry-jet wet spinning air gap and cold and hot drawing stages to achieve high orientation of BNNTs and a higher draw ratio of 25x. These fibers can be used to produce thermally conductive, electrically insulating composites, which have significant applications in electronics. This approach is scalable and can also be used to produce high-performance neat BNNT fibers.

## Graphical abstract

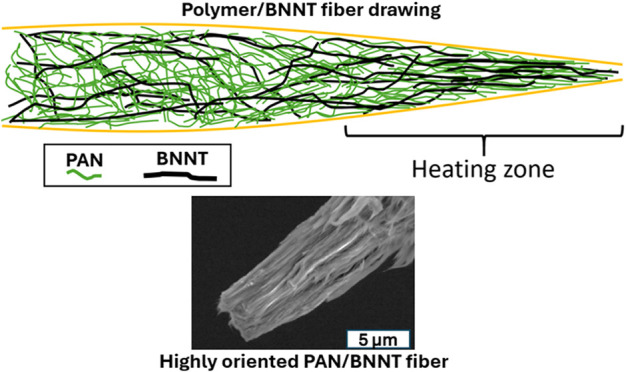

## 1 Introduction

Polyacrylonitrile (PAN) fibers have a wide range of applications, from clothing to carbon fibers. The first commercial production of PAN fibers was started by DuPont using solution spinning in the 1950s. PAN precursor fibers can be made by a variety of spinning techniques, including melt-assisted spinning (less common),^1,2^ dry spinning,^3^ wet spinning,^4^ and dry-jet wet spinning.^5^ PAN is commonly solution-processed because the polymer degrades before melting unless a solvent plasticizer is used.^1^ Solution processing using dry spinning, wet spinning, and dry-jet wet spinning requires the PAN polymer or its copolymer to be dissolved into highly polar solvents such as dimethyl sulfoxide (DMSO), *N*,*N*-dimethylformamide (DMF), and *N*,*N*-dimethylacetamide (DMAc).

PAN/carbon nanotube (CNT) composite fibers have been spun using dry-jet wet spinning,^6–8^ gel-spinning,^9,10^ electrospinning,^11,12^ and melt spinning.^13^ While PAN/CNT composite fibers have been extensively studied, far less attention has been directed toward analogous fibers featuring boron nitride nanotubes (BNNTs). There have been some reports of PAN/BNNT fibers, including two reports on gel-spinning,^14,15^ and one report on electrospinning.^16^ In both PAN/CNT and PAN/BNNT spinning, nanotubes remain dispersed in a suitable solvent for PAN, such as DMF or DMAc. Low nanotube loading in the fibers has drawability comparable to that of neat PAN fibers, but drawability decreases as more nanotubes are added. Higher drawing of fibers increases the polymer, nanotube, and pore alignment with the fiber axis and results in better tensile properties.^10,15^

The dispersion quality for fiber spinning depends on the nanotube characteristics (aspect ratio, number of walls, purity), polymer molecular weight, and interaction of the solvent with both polymer and nanotube. Typically, nanotubes are dispersed in the solvent first at very dilute concentrations (e.g., 0.005–0.6 mg nanotube/mL), before adding them to a prepared PAN solution and removing excess solvent. Extensive sonication and homogenization are used to obtain a stable nanotube dispersion,^8,10^ which often results in significant shortening of the nanotubes.^14^ While sonication times shorter than 1 h result in less damage to the nanotubes, their length still decreases.^17^ Some of the chief challenges arising from spinning of PAN/nanotube dispersions have been associated with shear-thinning, dispersion homogeneity, and elasticity and jet swelling. The increasing nanotube–nanotube interaction in the CNT network is shown to increase the shear-thinning behavior of PAN/CNT dispersions and the elasticity, which contribute to jetting issues.^6^ The rheological challenges of an interacting nanotube network make spinning PAN solutions with the addition of nanotubes quite complicated and difficult.

In this work, PAN/BNNT fibers containing long BNNTs are spun and drawn (Scheme 1) to study the effects of processing on the fiber structure–property relationships. Study and design of a fiber spinning process that preserves longer length BNNTs are important for further development of BNNT processing. These PAN/BNNT fibers are used as precursor fibers for BNNT fibers that are formed in subsequent work after removal of the polymer. These BNNT fibers are suitable for applications that require high thermal conductivity, high temperature oxidative resistance, and a low dielectric constant.

**Scheme 1:**
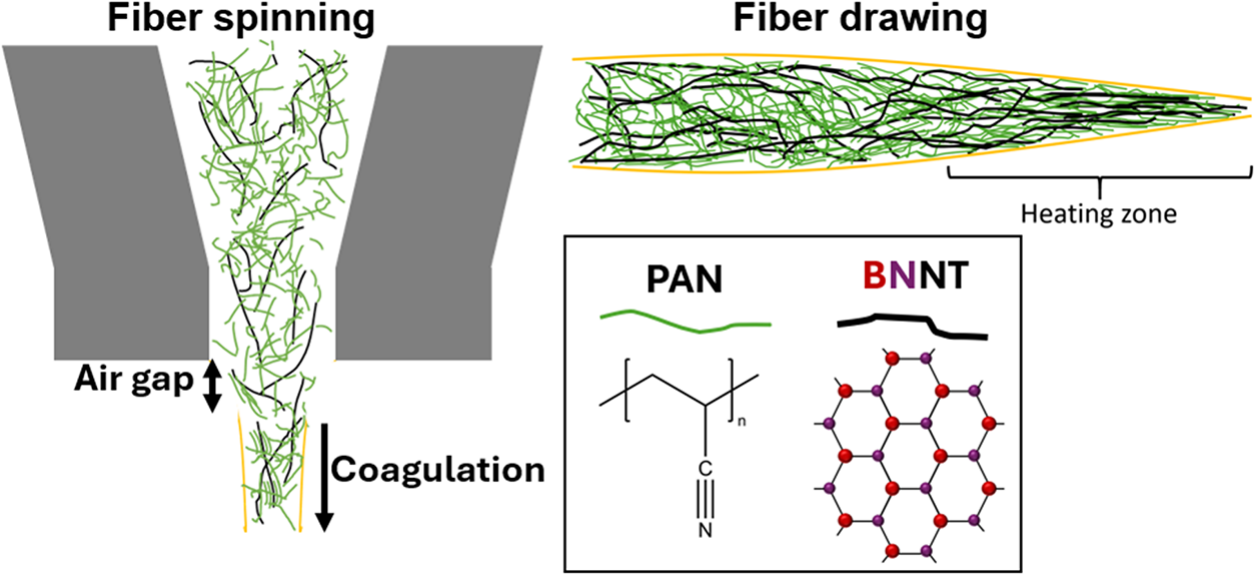
Graphical Depiction of the Spinning Process^a^

a PAN and BNNTs (in solvent) are extruded from a spinneret (typically with an air gap) before coagulating into a solid fiber (indicated by the gold surface of the fiber) and being drawn to align polymer chains and BNNTs.

## 2 Experimental Section

### 2.1 Materials

Polyacrylonitrile homopolymer [PAN, two molecular weights: 250 kg/mol (I) and 500 kg/mol (II)], copolymer containing 4 wt % methyl acrylate (PAN-*co*-MA, 510 kg/mol), and copolymer containing 4% methacrylic acid [PAN-*co*-MAA, two molecular weights: 500 kg/mol (I) and 986 kg/mol (II)] were obtained from Japan Exlan Co. Poly­(methyl methacrylate) (PMMA) was purchased from Millipore-Sigma-Aldrich (996 kg/mol). Boron nitride nanotubes were acquired from BNNT, LLC with a listed average length of 20 μm and a diameter of 3–5 nm. HPLC-grade dimethylacetamide (DMAc) was used as the solvent for both PAN and PMMA polymers and was used as-received from Millipore-Sigma. Methanol for the coagulation bath was used as-received and was procured from VWR.

### 2.2 Fiber Spinning

Table 1 lists the sample preparation and spinning conditions of the experimental trials conducted in this work. PAN/BNNT and PMMA/BNNT fibers were spun. In general, a dispersion of BNNT/DMAc with a concentration of ∼0.1 g BNNT/100 mL DMAc was sonicated, then added to a reactor with a predetermined polymer while stirring at a temperature of 1–2 °C. After 1 h stirring at low temperature, the temperature was increased to 90 °C, and then the set temperature was immediately decreased to 80 °C for excess solvent evaporation.

**Table 1:** List of Experimental Trials (T) and Their Process Conditions

	T1	T2	T3	T4	T7	T9	T10
polymer matrix	PAN-*co*-MAA, I	PAN-*co*-MAA, 20% I; 80% II	PMMA	PAN-*co*-MAA, 90% I; 10% II	PAN, II	PAN, I	PAN-*co*-MA
BNNT (wt % of polymer used)	∼10 wt %	∼20 wt %	∼5 wt %	∼5 wt %	∼5 wt %	∼5 wt %	∼5 wt %
sonication (mins)	30	30	30	30	120	120	40
dispersion-centrifuge cycle at 5000*g*	0	0	0	0	1	1	2
as-spun draw ratio	2.5	2	1.2	2	2.3	1.5	2.3
max. cold-draw ratio	-	-	-	-	1.25	1.25	1.25
max. hot-draw ratio	-	-	2	10	7	11	9
hot-draw temperature (°C)	-	-	110	150	150	150	150
total draw ratio	2.5	2	2.4	20	20	20	25

After the addition of the dilute BNNT dispersion to the PAN solution, the PAN/BNNT dispersion was evaporated until a suitable fiber-forming tendency was achieved. Once the solution reached a suitable state for spinning, a syringe spinning setup was used to spin fibers into a methanol/DMAc (80/20 v/v) coagulation bath at room temperature. This setup allowed for continuous dry-jet wet spinning using a spinneret syringe with a diameter of 260 μm. Fiber drawing was conducted in a glycerol bath in the temperature range 140–170 °C. Table 1 summarizes the materials, dispersion, and spinning and drawing conditions of the different trials.

### 2.3 Characterization

Dynamic light scattering (DLS), using a Wyatt DynaPro NanoStar, was conducted on the dispersed sediment and supernatant separated during centrifugation. Raman spectroscopy at 785 nm wavelength was conducted to determine the relative h-BN content in BNNT samples using a Renishaw Qontor Confocal Raman Microscope. Additionally, Raman Spectroscopy at a 266 nm wavelength was conducted on PAN/BNNT fibers using a Renishaw inVia UV Raman Microscope. Rheology of the PAN/BNNT dispersion was studied by using an ARES rheometer with a 50 mm diameter parallel plate geometry. The morphology of the fiber was captured using a Hitachi SU8230 scanning electron microscope (SEM) at an accelerating voltage of 5 kV. Individual fiber cross sections were measured using ImageJ for every trial, and at least 10 images were used to calculate the average diameter of the fibers. The tensile properties of the polymer/BNNT precursor fiber were determined using a Rheometrics solids analyzer, RSAIII. For the precursor fiber, single-fiber tensile tests were conducted at a gauge length of 12.5 mm and a strain rate of 1% per second. Wide-Angle X-ray Diffraction (WAXD) of the precursor fiber bundle was carried out using Rigaku Micromax 003 (Cu Kα, λ = 1.542 Å, 60 mA, 50 kV). X-ray exposure time was 1–2 h for each sample. The diffraction patterns were analyzed by using AreaMax and MDI Jade 9.1 software. The azimuthal full-width at half-max (FWHM) was determined from the (200,110) plane azimuthal scan at 2θ ≈ 17° for PAN, and the (200) plane azimuthal scan at 2θ ≈ 26° for BNNTs using the procedure previously described by Sreekumar et al.^8^ Transmission electron microscopy (TEM) was performed at NASA Glenn Research Center after sample preparation using focus ion beam (FIB) milling. TEM foils were FIB milled out from the fibers by using a ZEISS Auriga 40 dual-focused ion beam FIB-SEM with a Ga ion source. The FEI Talos F200S scanning/transmission electron microscope was then used for microstructural analysis and high-resolution imaging and characterization. Ultraviolet–visible (UV–vis) spectroscopy was performed on fibers and nanotubes with a Cary 5000 UV–vis/NIR.

## 3 Results and Discussion

### 3.1 Effects of Dispersion Processing

Existing literature regarding BNNTs has used sonication followed by centrifugation to purify the material based on the difference in diffusion behavior of the nanotubes and hexagonal boron nitride (h-BN) in the solvent.^18^ While sonication is used to break up and disperse bundles of nanotubes, it may also reduce the length of the nanotubes. After sonication and centrifugation, dispersed nanotubes in the supernatant are separated from the poorly dispersed nanotubes and large bundles in the sediment. Longer nanotubes have more potential contact area for interactions between adjacent tubes in dispersion, as well as a longer length scale for interacting and entangling with other tubes into a bundle. This makes it possible to remove shorter, well-dispersed bundles in the supernatant and increase the fraction of longer nanotubes in the dispersion for fiber spinning. Additionally, longer nanotubes can experience greater force during drawing than smaller nanotubes, which will be necessary to overcome the greater entanglement that will result from the longer tubes.^19^

DLS was used to study the relative changes in BNNT dispersions in DMAc throughout the sonication-centrifugation procedure. The DLS results shown here are interpreted qualitatively, as the dispersions studied have poor uniformity and nanotubes are not individualized in a way that would allow for quantification of the nanotube characteristics.^20^ However, changes to the average BNNT bundle size after sonication-centrifuge treatments can readily be observed in the DLS data. Table S1 shows descriptions of the DLS samples tested from two large-scale batches of processing in trials TA and TB, respectively. Two types of samples are examined from before and after centrifuge steps—dispersions of sediments after mixing and the supernatants removed after centrifuging.

Figure 1 shows the DLS data collected on supernatants from the first centrifuge (S1—lighter colors) and second centrifuge cycles (S2—bolder colors). The data shows that S1 supernatants have a smaller average size as well as a narrower range of sizes removed. The S2 supernatants contain sizes with an average radius of hydration (*R*_H_) ∼ 40 nm higher and with ∼2 nm higher standard deviation. As shown by red lines in Figure S1a, removal of S1 leads to a reduction in the bundle population around *R*_H_ = 105 nm, while removal of S2 leads to a broad reduction in the size distribution around *R*_H_ = 150 nm. Additionally, subsequent rounds of centrifugation remove the majority of the peak signal below 50 nm, showing more removal of the smaller structures using this centrifuge method. Data from repeated trial B (TB) is shown in Figure S1b.

**Figure 1:**
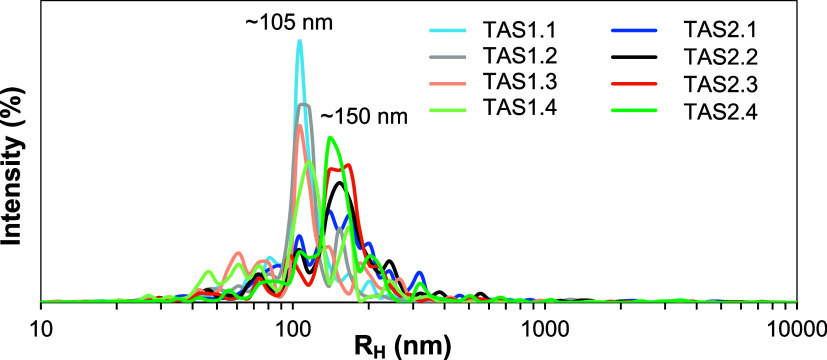
DLS of TA step 1 (S1) and step 2 (S2) supernatants after four identical centrifuge repetitions.

Sonication in solvent alone generally leads to a more polydisperse nanotube profile.^20^ Figure S2 shows that BNNT bundles are dispersed, and the DLS size distribution broadens at short sonication times <1 h, but longer times result in a rise in higher bundle size with *R*_H_ ∼ 250 nm. Large bundles may be forming after sonication due to reaggregation of the nanotubes in the absence of a surfactant. For this reason, sonication times of the BNNT dispersion were shortened from 2 h in trials T7 and T9 to 40 min sonication time for trial T10. Despite these changes, the overall dispersion quality of BNNTs in DMAc is still poor. Significant inhomogeneity is still present as clumps of BNNTs in the solvent and leads to irregular pouring from the bottle when combined with the PAN solution.

There are assumed to be essentially three constituents of the dispersion studied: h-BN, short BNNTs, and long BNNTs. Fourier-transform infrared spectroscopy (FTIR) and Raman are used to determine whether the removal of the supernatant significantly changes the relative content of h-BN vs nanotubes in dispersion. FTIR data shown in Figure S3 suggests that the h-BN content relative to the nanotube content of the as-received material is similar to the relative h-BN content after sonication-centrifuge. The conclusions of the FTIR data were replicated using 785 nm Raman (Figure S4), which also has been used to compare the relative amounts of h-BN in BNNT samples.^21–23^ This confirms that h-BN is not selectively removed over the shorter nanotubes during processing.

After BNNTs undergo a sonication-centrifuge procedure, the final BNNT dispersion in DMAc is added to a solution of dissolved PAN in DMAc. Agglomeration during this step is thought to be partially mitigated by incorporation into a dilute polymer solution before being concentrated for spinning. As the BNNT dispersion is added, the solution is evaporated until it reaches a suitable viscosity and fiber-forming tendency. PAN/BNNT precursor fibers are spun into a methanol or methanol/DMAc coagulation bath at room temperature, followed by drawing. The PAN/BNNT dispersion in its dilute state is shown in Figure 2a, while approximately 200-m spools of drawn T7.1 fiber are shown in Figure 2b.

**Figure 2:**
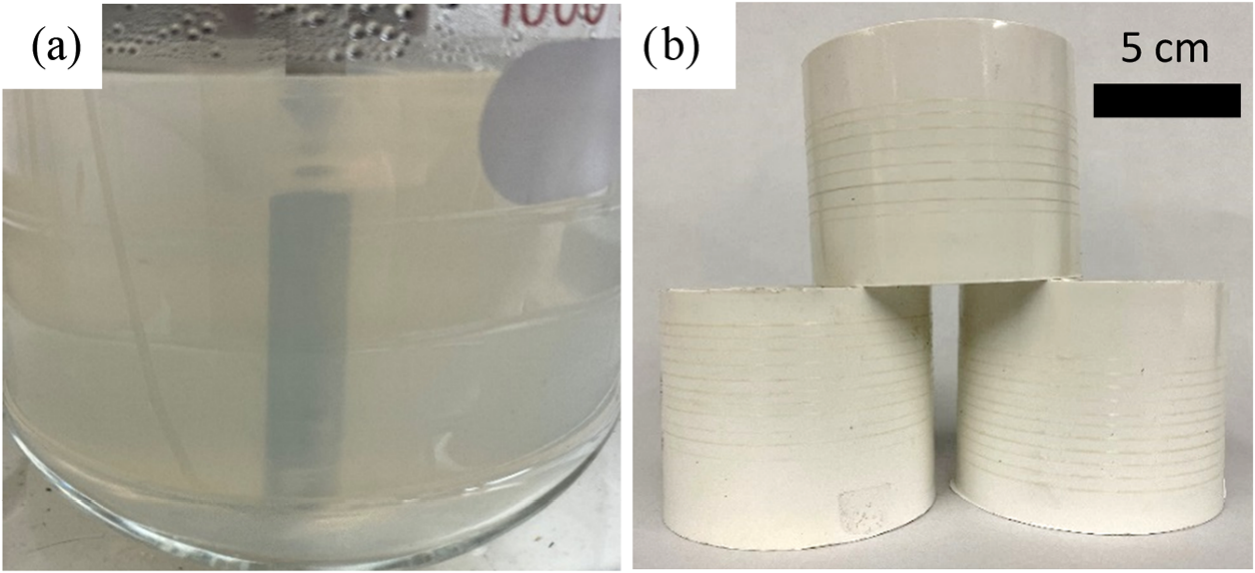
(a) Dilute dispersion of BNNTs in PAN during the addition/evaporation step. (b) T7.1 precursor fiber spools.

The rheology of the PAN/BNNT dispersion is important to the jetting behavior and spinnability at a larger scale. Rheological trends seen in earlier work by Lu et al. were observed here.^6^ Figure S5a–c show the rheological behavior of different trials. The high elasticity of the nanotube/PAN dispersion disrupted the good jetting behavior, which was remedied by lowering the polymer molecular weight (T9). For T4, T7, and T10 fiber trials, jetting was successful using a syringe pump spinning setup at a low flow rate (∼0.1 mL/min). When T7 dispersion was used in the Hills, Inc. spinning setup, described in earlier work,^6^ jetting and continuous fiber spinning became difficult. The challenges encountered with the larger-scale spinning setup likely originate from the presence of a filter and the challenging air gap control. It is believed that longer nanotubes and larger bundles are more likely to clog the system and be caught by the filter. The air gap should be less than 25 mm, as a longer air gap thins the fiber and causes breakage before collection.

### 3.2 Fiber Drawing and Alignment

In this study, drawing was performed in two stages: cold drawing at room temperature and hot drawing at temperatures >150 °C. Figure S6 shows the WAXD plate images, integrated scans, and PAN/BNNT azimuthal scans for the fibers. Cold drawing is conducted by small (∼1.25–2x) increases in the draw ratio and stretching of the fiber while it is at room temperature. For all samples, PAN crystal size was calculated using Scherrer’s equation, and crystallinity was calculated using the area ratio of the fitted crystalline peaks to the total area including an amorphous peak fitted at 2θ = 18°. Adjacent crystals are pulled together during drawing and crystal size increases (Table 2), but no morphological crystal change occurs. Hot drawing of the cold-drawn fiber is then conducted by passing the fiber through a glycerol bath at 150 °C at a high draw ratio (7–11x), increasing the crystal size and crystallinity. This rapid increase in ductility above ∼150 °C is ascribed to a reversible orthorhombic to hexagonal crystal/crystal transition, above which the molecular motion in crystalline regions is activated.^24,25^ A maximum draw ratio of 110× is possible for pure PAN fibers under optimum conditions.

**Table 2:** Structural Parameters and Tensile Properties of PAN/BNNT Precursor Fibers

sample ID	T1.0	T2.0	T4.0	T4.1	T4.2	T7.1	T9.1	T10.1	T10.2
total draw ratio	2.5	2.3	2.0	10	20	20	20	20	25
BNNT content (wt %)	10%	20%	5%	5%	5%	5%	5%	5%	5%
PAN crystallinity	37%	49%	41%	51%	59%	63%	71%	57%	65%
PAN_(200,110)_ crystal size (nm)	3.4	3.8	3.0	8.4	8.5	8.9	9.0	8.7	10.6
azimuthal FWHM_PAN(17°)_	71°	99°	86°	21.4°	15.8°	11.8°	9.2°	12.2°	9.2°
azimuthal FWHM_BNNT (26°)_			87°	16.2°	13.6°	10.1°	8.5°	10.8°	8.2°
diameter (μm)	48 ± 3	33 ± 3	38 ± 2	29 ± 2	13 ± 2	15 ± 2	16 ± 1	17 ± 1	12 ± 1
tensile strength (MPa) [max]	85 ± 36 [107]	54 ± 17 [96]	36 ± 13 [54]	207 ± 27 [252]	327 ± 47 [395]	438 ± 30 [486]	678 ± 46 [777]	427 ± 26 [508]	756 ± 46 [808]
modulus (GPa) [max]	4.6 ± 0.4 [5.4]	6.4 ± 0.8 [5.4]	4.4 ± 0.9 [5.4]	8.2 ± 0.6 [9.3]	9.4 ± 1.9 [13]	13 ± 1.3 [14]	19 ± 1.0 [22]	12 ± 0.9 [14]	19 ± 1.5 [22]
elongation to break (%) [max]	3.2 ± 1.2 [5.1]	1.2 ± 0.2 [1.5]	1.1 ± 0.3 [1.5]	7.6 ± 1.2 [8.9]	8.9 ± 2.6 [15]	9.1 ± 0.9 [11]	8.8 ± 1.0 [11]	9.0 ± 0.6 [10]	7.9 ± 1.6

Increasing the total draw ratio is shown to increase the alignment of the BNNT and PAN, as shown in Table 2. The increase in crystal size, crystallinity, and alignment led to an increase in the tensile properties of the drawn fibers. These results are consistent with earlier PAN/BNNT fiber literature by Chang et al.^14,15^ Additionally, there is a considerable jump in the drawn fiber properties of T7.1, in which a single centrifugation step was used to purify BNNTs. This advantage increased further as a second round of centrifugation was performed before spinning in the T10 trials. Table 2 shows the structure and properties of some of the PAN/BNNT precursor fibers.

Typical drawn PAN/nanotube fiber properties hover around 1 GPa strength and 20 GPa modulus.^10,15^ The fibers produced here are slightly weaker but show higher nanotube alignment. Figure 3a shows how the stress–strain curve of drawn precursors T4.1, T4.2, T7.1, T9.1, T10.1, and T10.2 compared to that of undrawn precursor T4.0. After tensile testing, BNNTs pulled out from the fiber surface (Figure 3b). There is a strong trend between the PAN/BNNT fiber mechanical properties and the orientation of both PAN and BNNT species within the fiber. The molecular orientation within the PAN/BNNT fiber was studied using the FWHM of the azimuthal scan at 2θ ∼ 17° for PAN and 2θ ∼ 26° for BNNTs, respectively.

**Figure 3:**
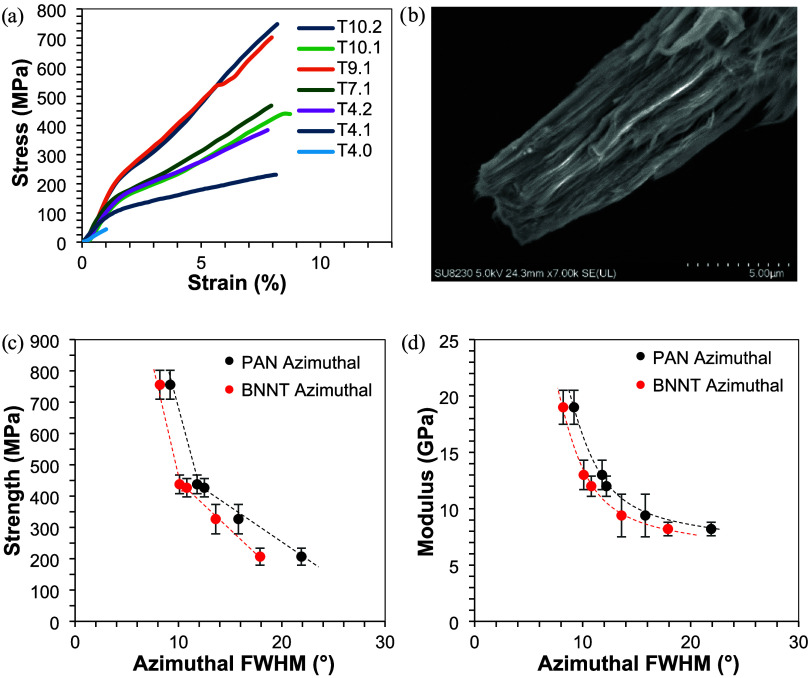
(a) Stress–strain behavior of as-spun and drawn PAN/BNNT precursor fibers, (b) SEM of T4.2 fiber after tensile testing; T4.1, T4.2, T7.1, T10.1, and T10.2 fibers, (c) tensile strength, and (d) modulus as a function of FWHM of the azimuthal scan of the 2θ ∼ 17° PAN (black) and ∼26° BNNT (red) WAXD peak.

While strength depends highly on defect size, the modulus is more dependent on the alignment of the underlying species. Figures 3c and 4d show the relationship between the orientation of the fiber, from the inverse relationship with FWHM, and the fiber strength and modulus, respectively. As the azimuthal FWHM decreases and the orientation of BNNT and PAN with the fiber axis increases, tensile strength increases bilinearly, and tensile modulus increases exponentially as the FWHM decreases below 10°. This is consistent with other work showing a substantial increase in the fiber modulus as the FWHM goes below 10°.^26^ While the trend of modulus is similar between the PAN and BNNT azimuthal FWHM, the trend of strength at lower orientations is slightly different. There is a greater increase in PAN orientation than in BNNT orientation at the same strength level. However, the strength versus orientation relation between PAN and BNNT is similar once orientation is increased. At low orientation, BNNTs act as defects and disrupt PAN’s crystalline structure, which is responsible for the PAN/BNNT fiber strength, whereas upon drawing, the BNNTs and PAN crystals are aligned together, and this disruption is minimized.

**Figure 4:**
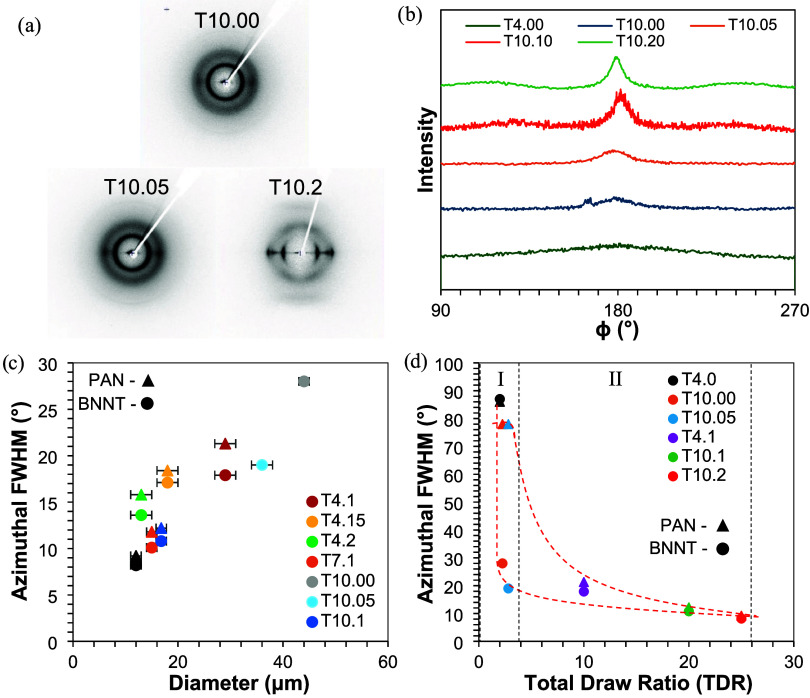
(a) Plate images of as-spun, cold-drawn, and hot-drawn T10 fibers; (b) azimuthal scans at 2θ ∼ 26° of T4 wet spun fibers and T10 dry-jet spun fibers as-spun, cold-drawn, and hot-drawn; (c) PAN/BNNT fibers azimuthal FWHM vs diameter relationship; (d) impact of drawing and drawing stages on alignment of PAN vs BNNT within the fiber.

After drawing, there is an increase in BNNT alignment from as-spun (whose azimuthal scan at 2θ ∼ 26° showed a peak intensity too low to measure) to a total draw ratio of 10×, 20×, and 25× (Figure 4b). Higher BNNT orientation in drawn PAN/BNNT fibers has also been confirmed in earlier work.^15^ When the fiber is drawn, the PAN (200,110) peak signal becomes more concentrated around the equatorial plane, similar to the BNNT (200) peak.

While cold drawing contributes to the BNNT orientation through high stress deformation, hot drawing contributes to the BNNT orientation through high flow deformation. Both processes lead to an increase in the BNNT orientation. During spinning with the syringe setup, the shear rate is relatively low (∼10^2^ s^–1^), which is likely not high enough to result in significant orientation.^27^ By studying the impact of the spinning setup, this work finds that a significant portion of the BNNT orientation occurs during extensional flow in the spinning air gap, with cold and hot drawing leading to further incremental alignment.

T4.0 as-spun fiber with no air gap shows little BNNT orientation with a BNNT FWHM of 86°. Whereas, T10.0 as-spun fiber with an air gap of ∼2 cm shows significant BNNT orientation with a FWHM of 28°. The orientation increases further after cold and hot drawing, showing BNNT FWHM values of 19 and 8.2°, respectively. BNNTs can be difficult to align without the presence of a solvent.^28^ Before entering the coagulation bath, the air gap provides a region of stretching where solvent molecules represent most (∼90 wt %) of the fiber, allowing for higher rotational diffusivity of the BNNTs in the spinning extrudate. Figure 4d shows graphically how the PAN and BNNT orientation, measured by azimuthal FWHM, change during the processing as the draw ratio increases. Region I corresponds to the as-spun and cold-draw regions, where the total draw ratio of the fiber is still relatively low and the BNNT orientation increases the most. Region II corresponds to the hot-draw region. Here, the total draw ratio increases quickly, and the PAN undergoes the greatest increase in orientation. Multiple cold-draw ratios were performed, and each showed an increase in the BNNT orientation with little change in the PAN orientation.

Both stages of drawing play a distinct role in aligning BNNTs within the precursor fiber, but PAN alignment occurs only during hot drawing. Minimal orientation is imparted to PAN during spinning and cold drawing, as the PAN FWHM after spinning is typically ∼80–90°. Due to the fast relaxation time of PAN, stretching at temperatures below the crystallographic transition temperature does not result in permanent alignment. However, the BNNTs will remain oriented from both shear and extensional flow when entangled with the polymer in the fiber. During spinning, BNNTs undergo shear force from extrusion in the spinneret, followed by extensional flow in the air gap, leading to a much larger increase in orientation.

Additionally, improvements in orientation during hot drawing are primarily realized when the final fiber diameter approaches <20 μm. The as-received nanotubes from BNNT, LLC are documented to have an average length of around 20 μm, with few BNNTs approaching 100 μm in length. With a fiber diameter below 20 μm, the BNNTs may be confined in an area with a diameter lower than the straight-line length of an average BNNT. Confinement effects would increase as the diameter decreases further because the BNNT will have increasingly limited states of off-axis orientation within smaller diameter fibers. This effect is suggested in Figure 4c by showing the relation between the fiber diameter and the BNNT orientation from the azimuthal FWHM. As is evident, after the introduction of the air gap and cold drawing (T7 and T10), there is a boost to the BNNT orientation at lower diameters. Also, BNNT bundles with length >20 μm are observed on the surface of drawn precursor fibers with diameters ∼15 μm (Figure S7a,b). This shows that nanotubes can still entangle radially around the fiber.

### 3.3 Fiber Structure

The distribution (bundled or exfoliated) and structure of the BNNTs within the polymer fiber were studied to better understand how processing was affecting the fiber structure. Exfoliated nanotubes exhibit van Hove transitions, which are observed as absorption peaks in UV–vis at wavelengths characterizing the nanotube band gap. While these transitions are suppressed when the nanotubes remain bundled, the drawing of PAN/CNT fiber has been shown to exfoliate CNTs and separate tubes from bundles. However, van Hove transitions were only observed in PAN/CNT fibers after reaching a draw ratio of 51× with a relatively low amount of CNTs (1 wt %).^29^

The BNNT band gap and the van Hove transition peaks are a function of the characteristics of individual BNNTs (diameter, chirality, and the intertube coupling of BNNT bundles). BNNTs should exhibit van Hove transitions from exfoliation in the UV wavelength range 200–300 nm, although this has not been documented. The band gap of BNNTs is reported to be in the range of 4–5.5 eV, and van Hove transitions should be observed as 4 absorption peaks in the UV wavelength range.^30,31^ Figure 5 shows the UV–vis of as-received BNNTs (T-B4), undrawn fibers T4.0 and T10.0, cold-drawn fiber T10.05, and hot-drawn fiber T4.2 and T10.2. The as-received BNNTs appear to have absorption at 215, 245, 265, and 297 nm (Figure 5a, labeled with red lines). These absorption peaks appear in the T10 undrawn fibers (Figure 5b) and T10 cold-drawn fibers (Figure 5c), but not in T4 fibers. The absence of van Hove transitions in T4 fibers could be due to the lack of an air gap during spinning and reduced exfoliation, causing a broadening of these absorption peaks and a less prominent response. Interestingly, these absorption peaks shift once the T10 cold-drawn fibers are hot-drawn, and the total draw ratio reaches 25× (Figure 5d, labeled in black lines). This shift appears to be a shift from 215, 245, 265, and 297 nm (5.8, 5.1, 4.7, and 4.2 eV) to 222, 258, 280, and 312 nm (5.6, 4.8, 4.4, and 4.0 eV). This band gap drop in 0.2–0.3 eV is very interesting and is hypothesized to be due to increased bundling occurring during hot drawing. Intertube coupling and nanotube wall interaction from bundling BNNTs has been observed to reduce the band gap by around 0.1 eV.^31^

**Figure 5:**
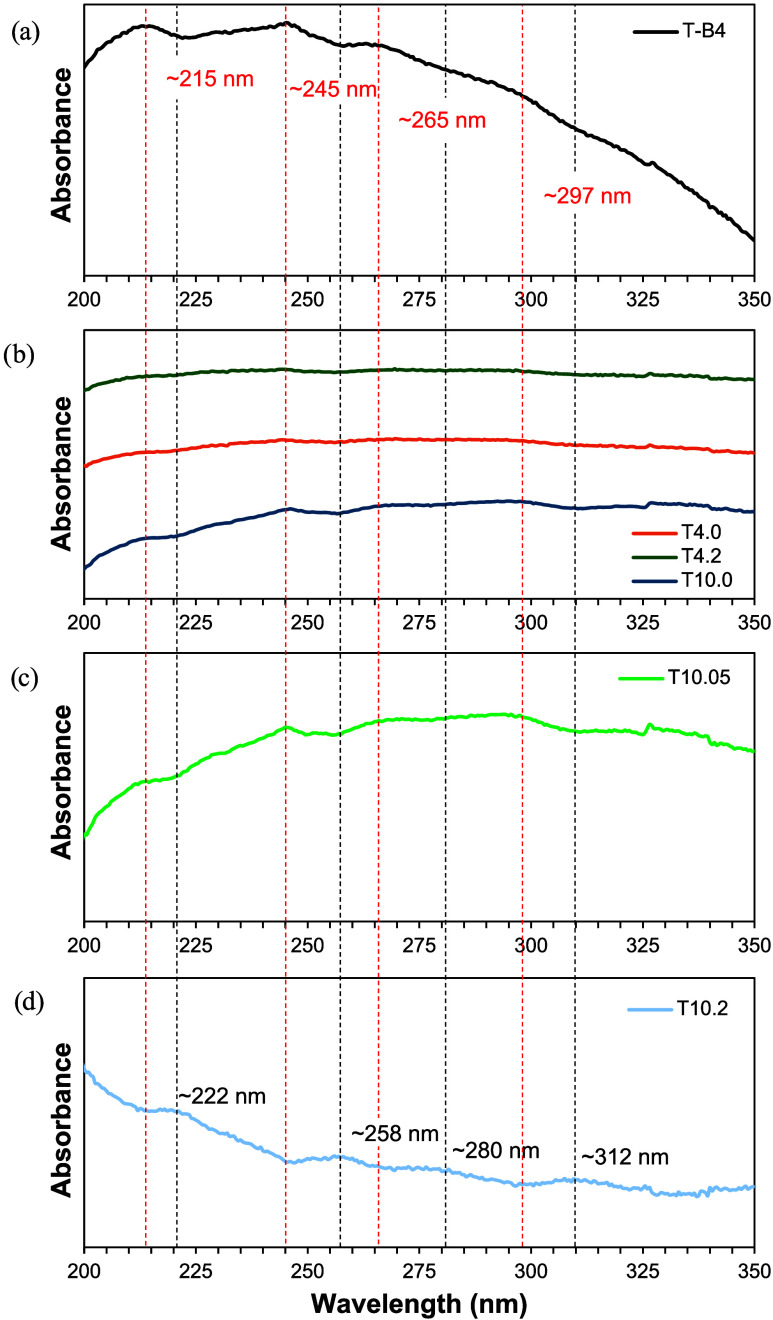
UV–vis spectra for samples (a) as-received T-B4 BNNTs and (b) T4.0, T4.2, T10.0, (c) T10.05, and (d) T10.2 PAN/BNNT fibers. Red lines indicate the position of the 4 peaks for the as-received BNNTs and black lines indicate the position of the shifted peaks in T10.2.

PAN/BNNT fiber studied using UV Raman supports the densification of the BNNT network after cold and hot drawing. After the fiber underwent cold and hot drawing, the response from the BNNTs at ∼1370 cm^–1^ increased. This could indicate that the BNNT network is becoming more densely packed relative to PAN within the Raman beam path. Cold drawing shows a moderate increase in the BNNT peak intensity without a substantial increase in the PAN peak intensity (1620 cm^–1^). In the case of hot drawing, both peaks of PAN and BNNTs become more intense, indicating that hot drawing is densifying both domains. Figure 6a shows the UV Raman spectra of PAN/BNNT fiber T10.00 (as-spun), T10.05 (after cold-draw), and T10.20 (after cold- and hot-draw). Additionally, the table in Figure 6b shows information for the Raman peaks of BNNT and carbon.

**Figure 6:**
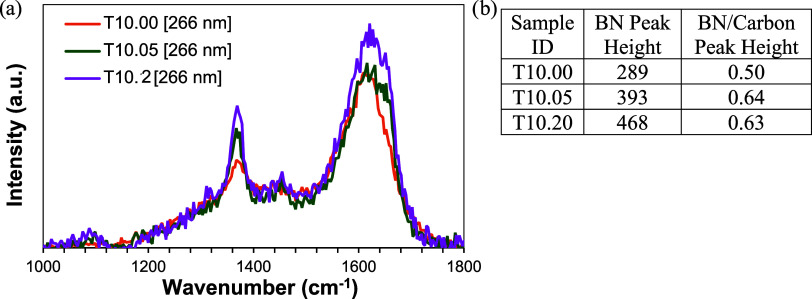
(a) 266 nm wavelength Raman spectra of undrawn T10 (T10.00), cold-drawn T10 (T10.05), and cold-drawn and hot-drawn T10 (T10.1) fibers. (b) Table of peak heights and ratios.

Bundled BNNTs can be viewed as crystallite domains within the polymer fiber. The UV Raman spectrum shows that the orientation increase from fiber drawing is also contributing to an increase in the density of the BNNT network in hot and cold drawing, albeit in different ways. At room temperature, cold drawing pulls the crystallites together along the fiber axis. The cold-draw ratio is limited to 1–2x, and the polymer network is less mobile, but adjacent BNNT crystallites are exfoliated and densified along their length. At a higher temperature of 150 °C, the polymer network becomes sufficiently mobile to reorganize and increase crystal size with a hot-draw ratio of 8–12x. Hot drawing densifies the BNNT crystallites along their diameter, increasing the BNNT wall-to-wall interaction within the crystallite and shifting the UV–vis spectra.

TEM was conducted on the T10 fiber to directly observe the BNNT structure within the fiber. TEM images in Figure 7 show that BNNTs take the form of independent channels of nanotubes aligned along the polymer fiber axis. These BNNT channels are well dispersed within the polymer fiber, as seen in Figure 7a,b. The nanotube count in each channel varies significantly. Channels with a larger number of BNNTs are seen in Figure 7c and smaller numbers of channels are shown in Figure 7d. However, the BNNTs largely exist as islands of multiple nanotubes, each well-aligned within the same channel (Figure 7e). Figure 7f also shows that defects in the polymer fiber can deflect the BNNT channels away from the fiber axis. These channels match the results of subsequent work, which showed that removal of the polymer creates fibrils of these BNNT channels that come together depending on the rate of polymer removal and the tension during removal.

**Figure 7:**
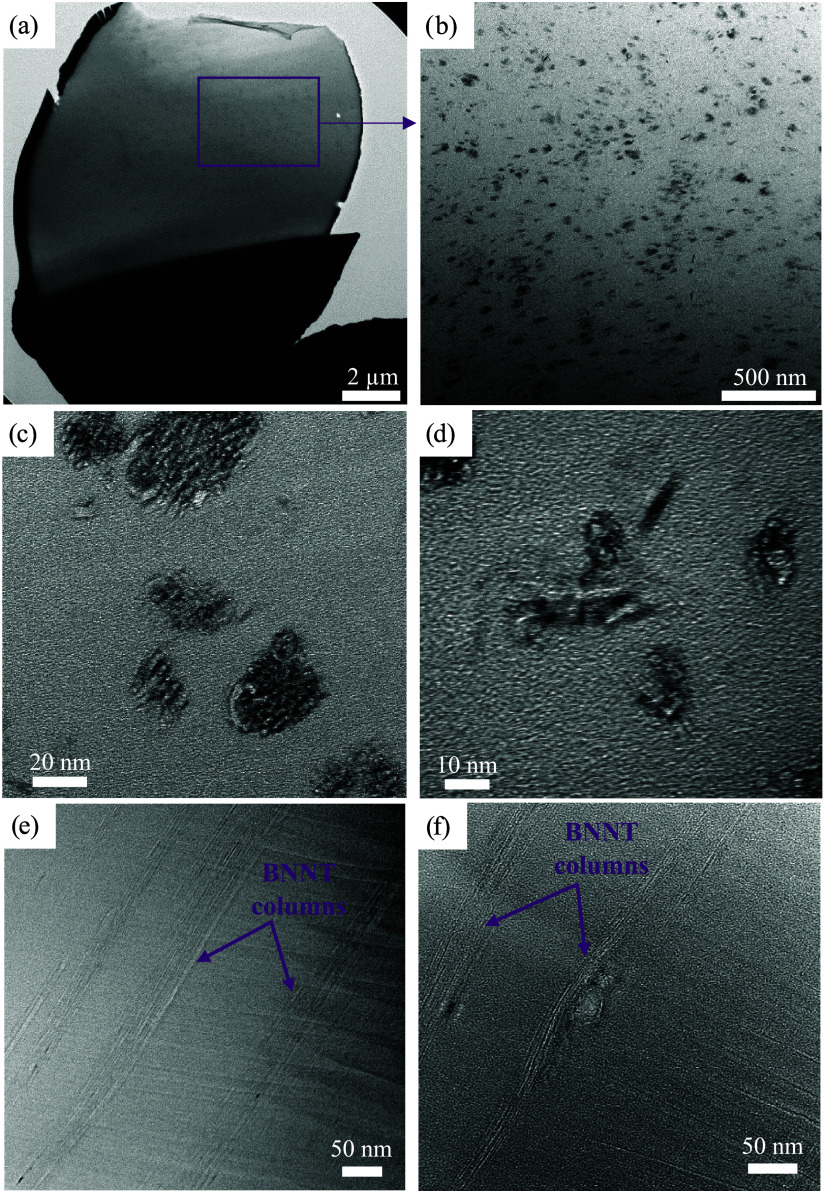
TEM images showing T10.2 fiber (a) cross-section with (b) good BNNT channel dispersion in PAN, and (c) channels with a large number of BNNTs, and (d) a small number of BNNTs; longitudinal section showing (e) noninteracting, independent BNNT channels, and (f) deflection of BNNTs around defects.

The observed UV–vis behavior offers a more complete explanation of the BNNT channels observed in TEM. Air gap spinning and cold drawing promote alignment of the BNNT channels along the fiber length, while hot drawing increases the density of the BNNT channels in the fiber cross-section. PAN mobility during hot drawing is significantly higher than the BNNTs, leading to densification of the BNNT network as adjacent channels are pulled together like crystallites. This observation is the opposite of what was seen in earlier PAN/CNT work,^29^ but this difference may be due to the extensive sonication time used in that work that created much shorter CNTs in a network with reduced potential for CNT-CNT entanglements and interaction within the fiber. During hot drawing, shorter nanotubes may be pulled past each other and exfoliated, while longer nanotubes have more entanglements and length to be pulled into each other.

## 4 Conclusions

Study and design of a fiber spinning process that preserves longer length BNNTs is important for further development of BNNT processing. In this work, entangled bundles of long BNNTs are created during sonication-centrifuge dispersion processing. The longer nanotubes align significantly during air gap spinning with the solvent present to facilitate mobility. Then, the high tension from cold drawing further orients the BNNT channels along the fiber axis. Finally, extensional flow during hot drawing densifies this structure, pulling adjacent BNNT channels together, while the polymer structure reorganizes at higher temperatures. This behavior is a result of differences in the force exerted on the BNNTs during the three drawing steps. After hot drawing, a BNNT column structure consisting of bundled BNNTs surrounded by polymer is observed.

Understanding the effects of alignment for longer BNNTs in a polymer matrix is important for processing these materials commercially. Follow-up work with these PAN/BNNT fibers sees the use of various heat treatment environments to remove the polymer and create BNNT fibers, as well as composite ceramic/BNNT fibers. Longer nanotubes have significant dispersion processing challenges that complicate commercial use, but longer nanotubes also have an advantage in performance and properties over shorter nanotubes in composites. The high thermal conductivity of nanotubes, combined with the strength, modulus, and electrically insulating properties, makes the incorporation of longer BNNTs into low-K dielectric composites a valuable direction to increase heat management capabilities in electronic applications.

## Supplementary Material



## Notes

DLS data of additional sediments and supernatants, FTIR and Raman of BNNT samples, rheology of PAN/BNNT dispersions, WAXD scans of PAN/BNNT fibers, and SEM images of fiber surfaces (PDF)
