# Supplementary material for: Processing–Structure–Property Relationships in Polymer/Boron Nitride Nanotube Composite Fibers for Electronic Packaging Applications
Source: ACS Appl Nano Mater. 2025 Sep 18;8(38):18274–83. doi: 10.1021/acsanm.5c02920 (PMC12481483; doi:10.1021/acsanm.5c02920)
Supplement: Supplementary file 1 [file an5c02920_si_001.pdf]

Supporting Information

Processing–Structure–Property Relationships in Polymer/Boron Nitride Nanotube Composite Fibers for Electronic Packaging Applications

Casey L. Smith[1], Keenan J. Mintz[1*], Kishor Gupta[1], Anita Garg[2,3], Laura Wilson[3], Satish Kumar[1]

1. Georgia Institute of Technology, School of Materials Science and Engineering; Atlanta, GA, 30332, USA
2. University of Toledo, Mechanical Industrial and Manufacturing Engineering; Toledo, Ohio, 43606, USA
3. NASA Glenn Research Center; Cleveland, Ohio, 44135, USA

*Corresponding author email: kmintz3@gatech.edu

Table S1: DLS samples from trials TA and TB

**Sample ID**	**Description**
TAS1.1	Trial A, supernatant 1 removed from 1st centrifuge cycle
TAS1.2	Trial A, supernatant 2 removed from 1st centrifuge cycle
TAS1.3	Trial A, supernatant 3 removed from 1st centrifuge cycle
TAS1.4	Trial A, supernatant 4 removed from 1st centrifuge cycle
TAS2.1	Trial A, supernatant 1 removed from 2nd centrifuge cycle
TAS2.2	Trial A, supernatant 2 removed from 2nd centrifuge cycle
TAS2.3	Trial A, supernatant 3 removed from 2nd centrifuge cycle

**Sample ID**	**Description**
TAS2.4	Trial A, supernatant 4 removed from 2nd centrifuge cycle
TAD1.5	1.2 mg/mL BNNT in DMAc dispersion after mixing
TAD2.5	TAD1.5 after 1st centrifuge cycle sediment redispersed
TAD3.5	TAD2.5 after 2nd centrifuge cycle sediment redispersed
TAS1.5	Trial A, supernatant 5 removed from 1st centrifuge cycle
TAS2.5	Trial A, supernatant 5 removed from 2nd centrifuge cycle

**Sample ID**	**Description**
TBD1.5	1.2 mg/mL BNNT in DMAc dispersion after mixing
TBD2.5	TBD1.5 after 1st centrifuge cycle sediment redispersed
TBD3.5	TBD2.5 after 2nd centrifuge cycle sediment redispersed

**Sample ID**	**Description**
TBS1.5	Trial B, supernatant 5 removed from 1st centrifuge cycle
TBS2.5	Trial B, supernatant 5 removed from 2nd centrifuge cycle

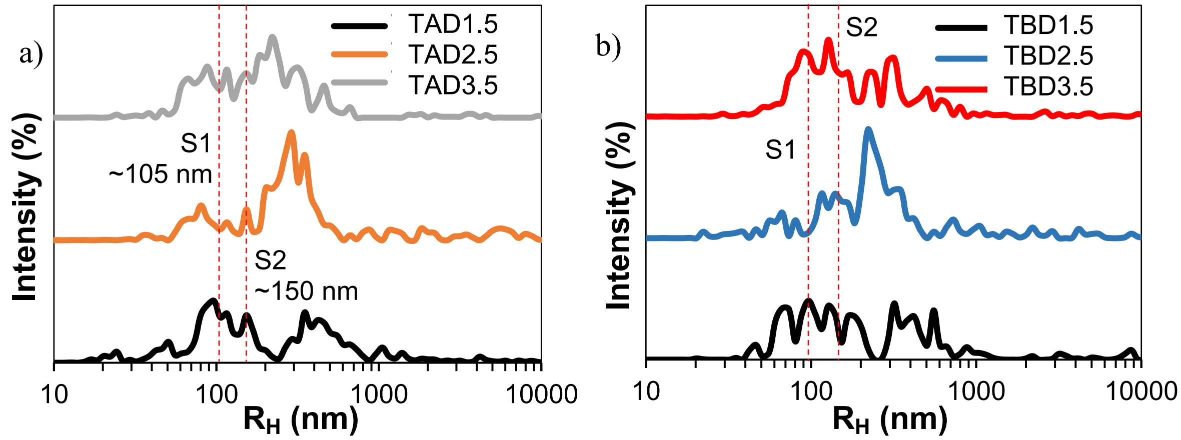

Figure S1: trial TB (a) sediment after centrifuge and redispersion, and (b) removed supernatants. Red lines indicate the main size from the removed supernatants.

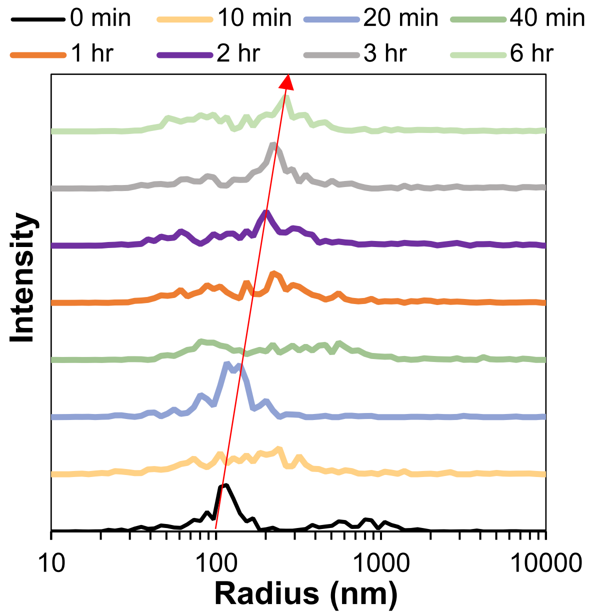

Figure S2: DLS of sonicated nanotubes as sonication time increase

From the data collected, the as-received BNNTs T-4 were compared with purified T-4 P nanotubes from the supplier as well as the sediment T-4 SC after the 2 cycle sonication-centrifuge procedure (Figure S3a). The h-BN content of these samples was compared using the area ratio and the intensity ratio of the out-of-plane (OP) B-N peak to the in-plane (IP) B-N peak [1], [2]. The table in Figure S3b shows the calculated OP/IP ratios for the samples using both area and intensity methods. T-4 P BNNTs, purified by the manufacturer to remove h-BN, show noticeably lower OP/IP ratios. As-received T-4 BNNTs and T-4 SC sonication-centrifuged BNNTs show similar OP/IP ratios, with the T-4 SC OP/IP ratio being ~10% higher. T-4 P BNNTs also have OP and IP peaks blue shifted to higher wavenumber, also indicating a reduction in h-BN content.

a)

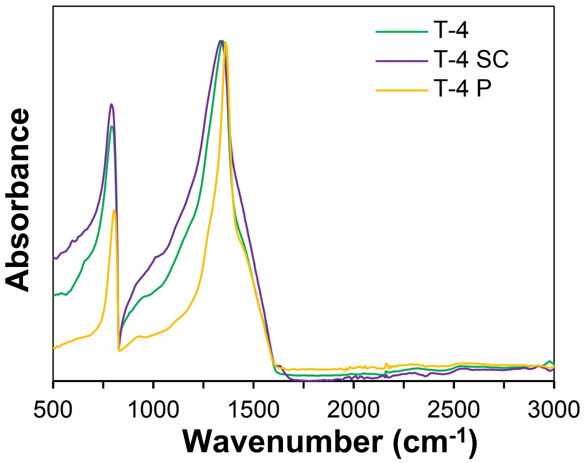

b)

Sample Code	OP/IP (Intensity)	OP/IP (Area)	OP (cm^{-1})	IP (cm^{-1})
T-4	0.75	0.46	795	1346
T-4 SC	0.81	0.52	791	1336
T-4 P	0.50	0.21	806	1362

Figure S3: (a) FTIR spectra of batch 4 BNNTs (T-4) used in fiber trials, T-4 BNNTs after sonication centrifuge procedure (T-4 SC), and purified BNNTs from BNNT, LLC (T-4 P); (b) table detailing the out-of-plane (OP) and in-plane (IP) BN characteristics of the samples, indicating relative h-BN amount

Using the method described in literature, the full-width at half maximum (FWHM) is compared between the BN peaks centered at 1365-1370 cm^{-1} [2], [3], [4]. The content of h-BN in the sample decreases as the FWHM of the BN peak increases. Additionally, samples with Raman peak blue shifted closer to 1370 cm^{-1} contain single-walled nanotubes. Figure S6a shows the Raman spectra of T-4, T-4 SC, and T-4 P compared to h-BN, where the peak width of the purified sample shows a noticeable increase. The table in Figure S6b shows the wavenumber of the BN peak center and FWHM of the BN peaks, which were calculated by fitting a Lorentzian peak to the data.

a)

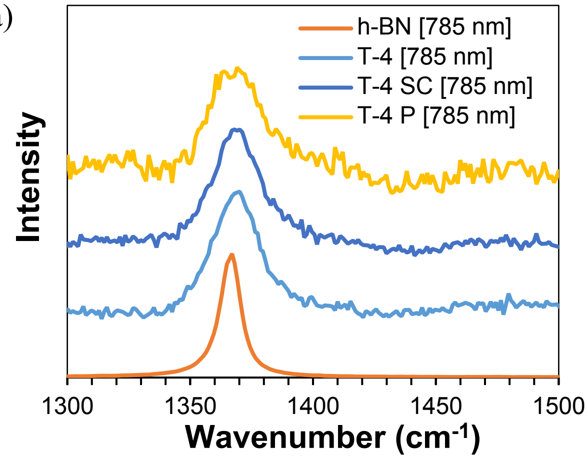

b)

Sample Code	Raman FWHM	BN peak (cm^{-1})
h-BN	9.7°	1366
T-4	23.6°	1368
T-4 SC	24.6°	1368
T-4 P	34.1°	1369

Figure S4: (a) 785 nm Raman spectra of T-4, T-4 SC, and T-4 P BNNTs; (b) table detailing the FWHM and peak center of the BN Raman peak for each sample

Figure S6b and S4c show the shear thinning behavior of T7 and drop in tanδ at high shear rate, where the high shear creates elastic forces that leads to swelling that disrupts good jetting behavior. Trial T9 was attempted with 250K molecular weight homopolymer, allowing for good jetting at >1 mL/min. While the T9 solution remains shear thinning, the drop in tanδ at high shear rates is not as pronounced due to higher inelastic forces present from the lower molecular weight PAN. The explanation for this behavior originally comes from Lu et al., where the lower molecular weight polymer allows for less elastic behavior of the dispersion and negatively affects the jetting [5].

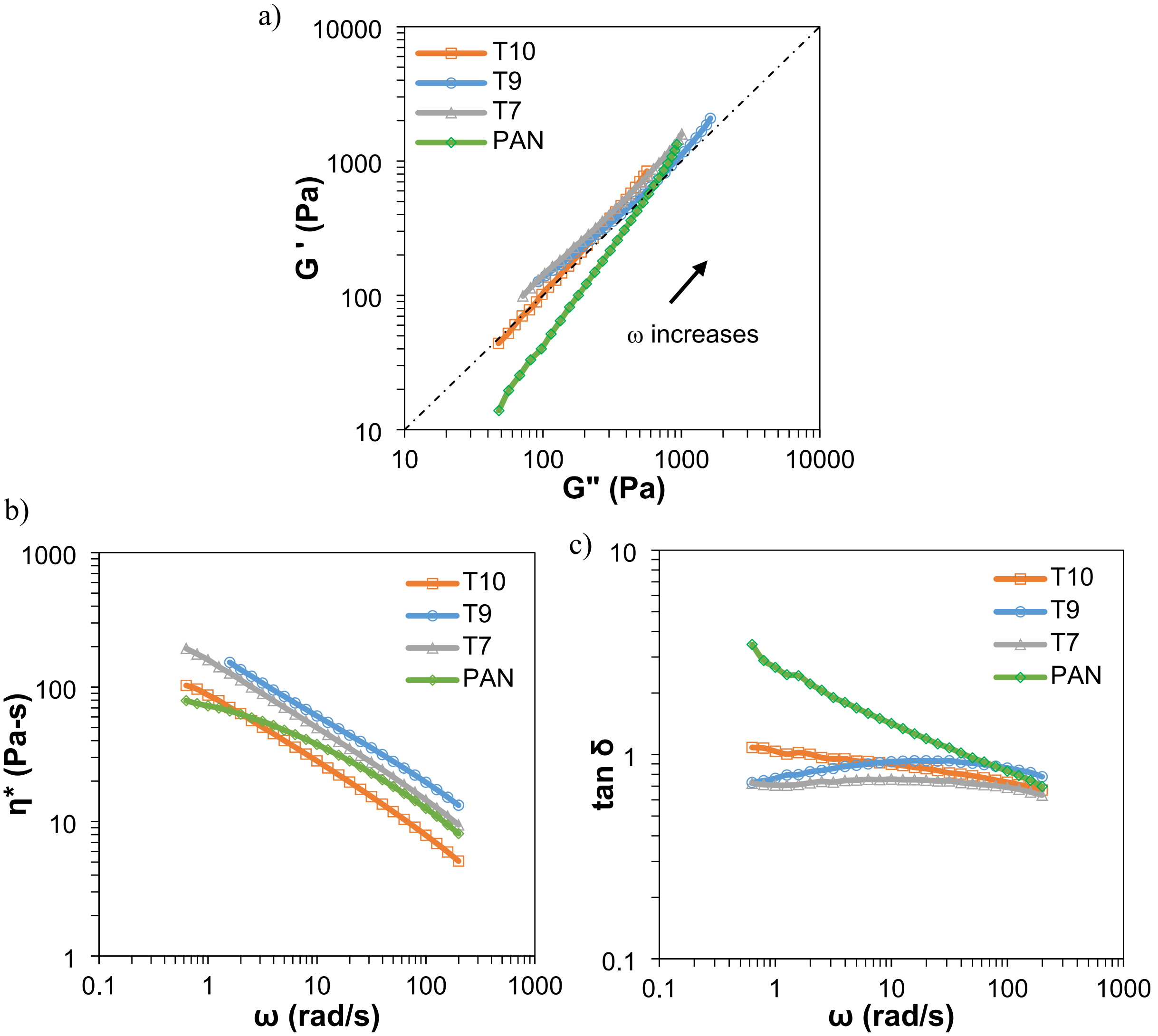

Figure S5: PAN/BNNT dispersions (a) log-log plot of G″ versus G′, (b) complex viscosity (η*) as a function of ω, (c) tan δ as a function of ω; data measured for all samples at 20 °C

WAXD of PAN fibers consists of two relatively sharp equatorial diffraction lines around 2θ ~ 17° (200) and 29° and two relatively broad lines from diffraction close to the meridian at 2θ ~ 25° and 40°. The crystal structure of PAN can be either hexagonal or orthorhombic, identified by the location of the 2θ ~ 17º peak. If the 2θ of the peak is higher and closer to 17°, the fiber has hexagonal crystal structure, which is the case for the precursor fiber here. Considering the crystal size of the as-spun fiber T1.0, T2.0, and T4.0 (3.0-3.8 nm) and drawn fiber T4.1, T4.2, T7.1, T9.1, T10.1 and T10.2 (8.4-10.6 nm), PAN crystal size and crystallinity increases after hot drawing the PAN/BNNT fiber. PAN orientation also increases from the as-spun fiber T1.0, T2.0, and T4.0 (FWHM of 71-86°) to the drawn fiber T4.1, T4.2, T7.1, T9.1, T10.1 and T10.2 (FWHM of 21-9.2°). BNNT orientation within the fiber also increases from the as-spun fiber T4.0 (FWHM of ~87°) to the drawn fiber T4.1, T4.2, T7.1, T9.1, T10.1 and T10.2 (FWHM of 16.2-8.2°). The plate images (Figure S6a), integrated scans (Figure S6b), and azimuthal scans of the fibers at 2θ ~ 17º (Figure S6c) and 2θ ~ 26º (Figure S6d) show these trends as the fiber is drawn more.

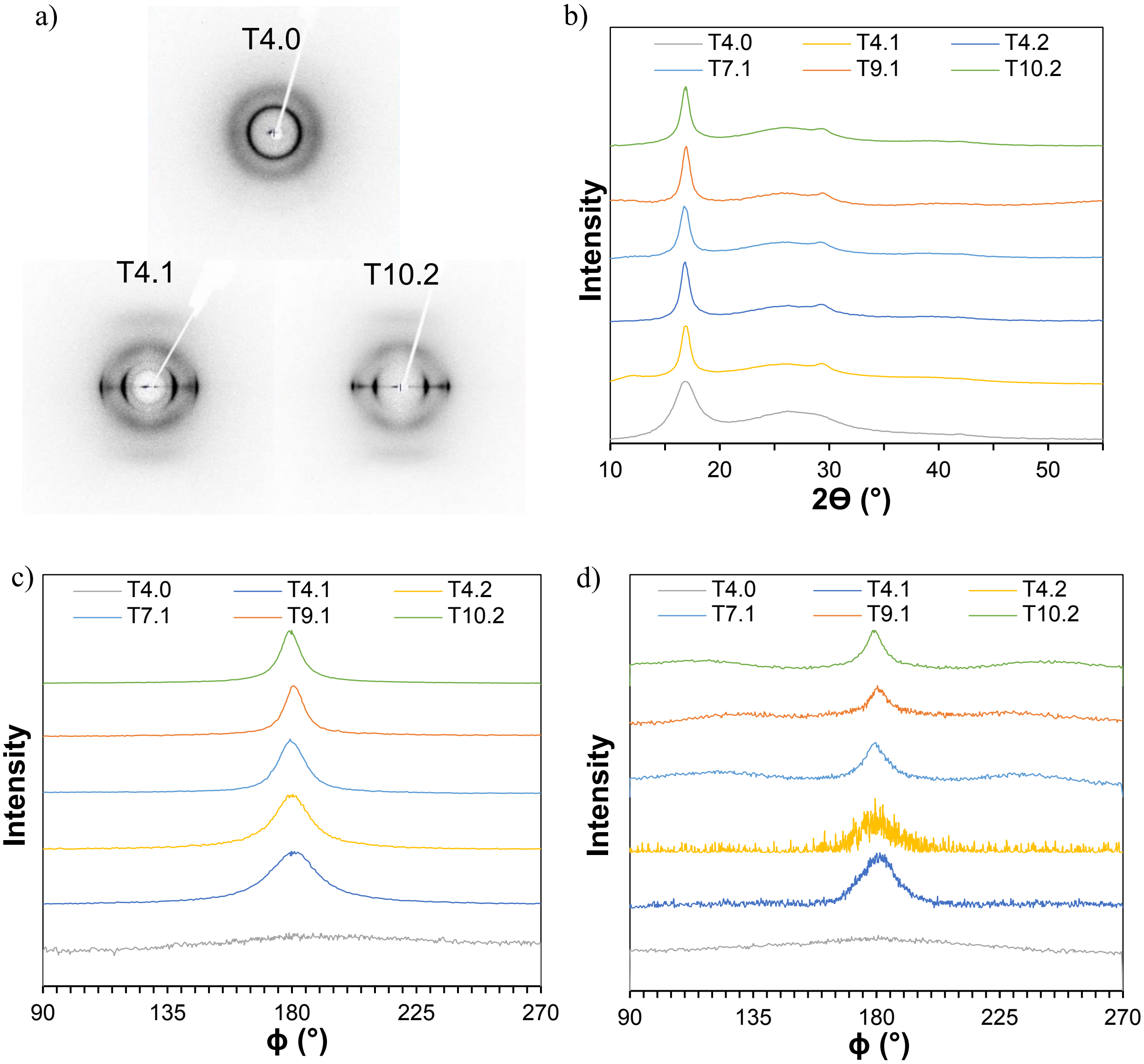

Figure S6: PAN/BNNT precursor fiber WAXD (a) plate images, (b) integrated scans, (c) PAN azimuthal scans (at 2θ = 17°), and (d) BNNT azimuthal scans (at 2θ = 26°)

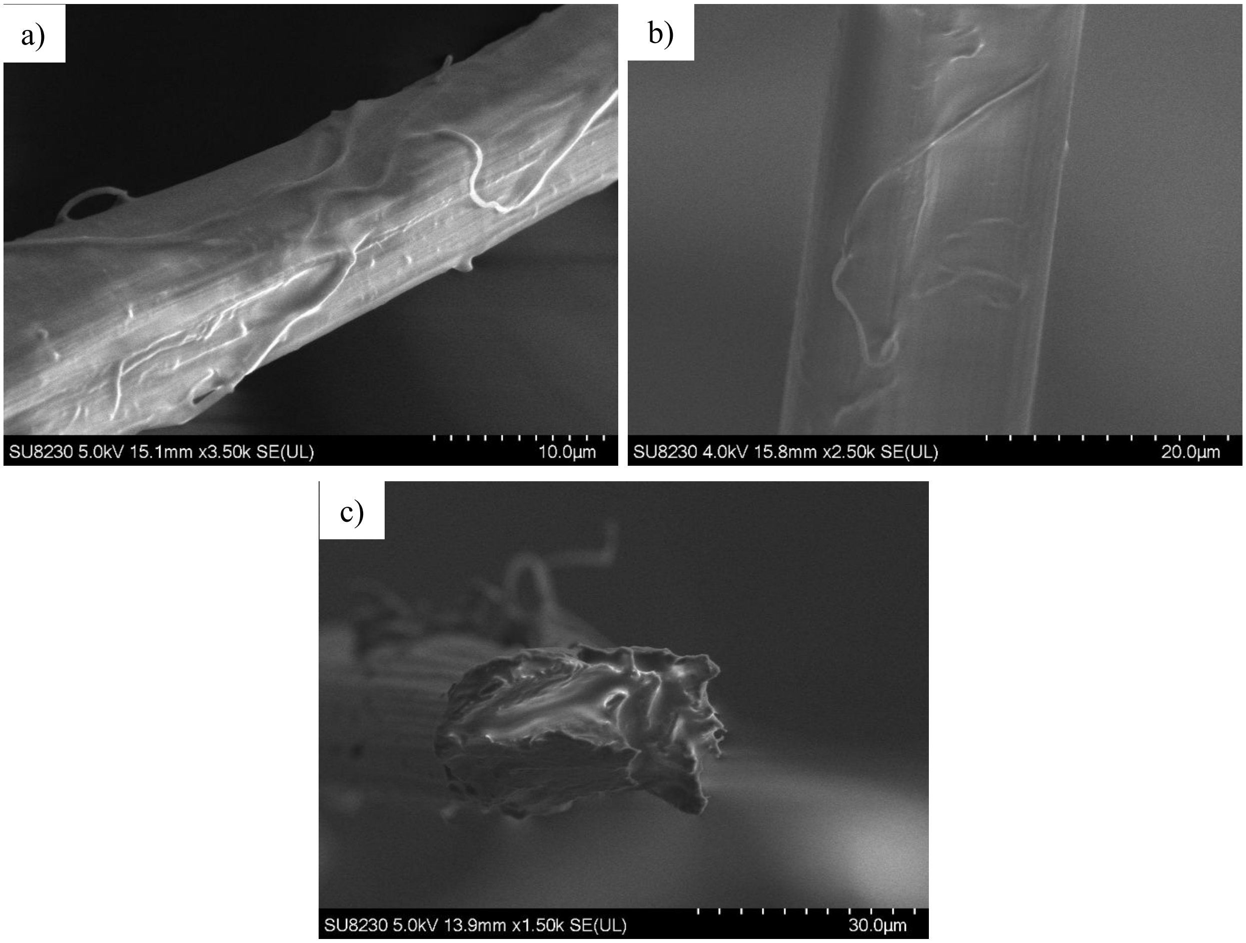

Figure S7: SEM of fiber surfaces of (a) T7.1, (b) T9.1, and (c) T4.0

Additional Discussion:

The tensile performance of T9.1 and T10.2 shows interesting behavior in figure 8a. While the processing of T9.1 and T10.2 are similar, T9.1 fiber shows a distinct stress relaxation at ~5% strain in 12/20 tensile tests. This relaxation is clear when compared directly with T10.2 fiber that has within 10% of the average tensile properties as T9.1, but stress relaxation was not observed in any samples. There are two changes made in trial T9 that may be responsible for this relaxation. First, fiber trial T9 used a 100 μm filter during spinning, which may remove some of the larger BNNT bundles within the dispersion. This removal then impacts the high-strain behavior of the PAN/BNNT fiber, as longer BNNTs are less present to load-share after a certain stretching distance. Second, fiber trial T9 used 250K g/mol molecular weight PAN instead of 500K g/mol PAN-co-MA (trial T10). As the strain distance at 5% vs 8% strain is ~700 μm vs ~1000 μm, PAN molecular weight is likely not coming into play at these length scales. Most likely, the removal of larger nanotube bundles is responsible for the stress relaxation at low strain.

Since torque is directly proportional to the distance of the force from the nanotube center, it follows that maintaining the same applied force and increasing the average nanotube length will lead to more torque on the nanotube and, therefore, more alignment. There are two effects that are likely to be at play—shear and velocity gradients that align the nanotubes during extensional flow

and confinement effects that further increase alignment with the fiber axis as the fiber diameter is reduced below the nanotube lengths [6], [7]. During cold drawing, the applied force in the fiber is relatively high and the structure improves by a mechanism similar to the strain hardening by plastic deformation. The amount of deformation to reach a stable neck represents the natural draw ratio. Ductile polymers form stable necking regions because molecular orientation provides a mechanism for hardening that predominates at large strains [8]. During hot drawing, the applied force on the fiber is lower as the fiber is drawn above glass transition temperature, but alignment by extensional flow is high.

References:

[1] Harrison, H. *et al.*, "Quantification of hexagonal boron nitride impurities in boron nitride nanotubes via FTIR spectroscopy," *Nanoscale Adv.*, vol. 1, no. 5, pp. 1693–1701, 2019, doi: 10.1039/C8NA00251G.

[2] Amin, M. S., Molin, T. E., Tampubolon, C., Kranbuehl, D. E., and Schniepp, H. C., "Boron Nitride Nanotube Impurity Detection and Purity Verification," *Chem. Mater.*, vol. 32, no. 21, pp. 9090–9097, Nov. 2020, doi: 10.1021/acs.chemmater.0c03609.

[3] Ko, J., Kim, H. M., Moon, S. Y., Ahn, S., Im, S. G., and Joo, Y., "Highly Pure, Length-Sorted Boron Nitride Nanotubes by Gel Column Chromatography," *Chem. Mater.*, vol. 33, no. 12, pp. 4723–4732, Jun. 2021, doi: 10.1021/acs.chemmater.1c01165.

[4] Amin, M. S., Atwater, B., Pike, R. D., Williamson, K. E., Kranbuehl, D. E., and Schniepp, H. C., "High-Purity Boron Nitride Nanotubes via High-Yield Hydrocarbon Solvent Processing," *Chem. Mater.*, vol. 31, no. 20, pp. 8351–8357, Oct. 2019, doi: 10.1021/acs.chemmater.9b01713.

[5] Lu, M. *et al.*, "Rheological behavior and fiber spinning of polyacrylonitrile (PAN)/Carbon nanotube (CNT) dispersions at high CNT loading," *Polym. (Guildf)*, vol. 215, p. 123369, 2021, doi: https://doi.org/10.1016/j.polymer.2020.123369.

[6] Rosén, T., Mittal, N., Roth, S. V, Zhang, P., Lundell, F., and Söderberg, L. D., "Flow fields control nanostructural organization in semiflexible networks," *Soft Matt.*, vol. 16, no. 23, pp. 5439–5449, 2020, doi: 10.1039/C9SM01975H.

[7] Calabrese, V., Haward, S. J., and Shen, A. Q., "Effects of Shearing and Extensional Flows on the Alignment of Colloidal Rods," *Macromolecules*, vol. 54, no. 9, pp. 4176–4185, May 2021, doi: 10.1021/acs.macromol.0c02155.

[8] Ziabicki, Andrzej., *Fundamentals of fibre formation : The science of fibre spinning and drawing* London ; SE - xiv, Wiley, 1976.
